# Parathyroid Hormone Fragments: New Targets for the Diagnosis and Treatment of Chronic Kidney Disease-Mineral and Bone Disorder

**DOI:** 10.1155/2018/9619253

**Published:** 2018-11-29

**Authors:** Huimin Chen, Xiaxia Han, Ying Cui, Yangfan Ye, Yogendranath Purrunsing, Ningning Wang

**Affiliations:** ^1^Department of Nephrology, The First Affiliated Hospital of Nanjing Medical University, Jiangsu Province Hospital, Nanjing, Jiangsu Province 210029, China; ^2^Nanjing Medical University, Nanjing, Jiangsu Province 211166, China

## Abstract

As a common disorder, chronic kidney disease (CKD) poses a great threat to human health. Chronic kidney disease-mineral and bone disorder (CKD-MBD) is a complication of CKD characterized by disturbances in the levels of calcium, phosphorus, parathyroid hormone (PTH), and vitamin D; abnormal bone formation affecting the mineralization and linear growth of bone; and vascular and soft tissue calcification. PTH reflects the function of the parathyroid gland and also takes part in the metabolism of minerals. The accurate measurement of PTH plays a vital role in the clinical diagnosis, treatment, and prognosis of patients with secondary hyperparathyroidism (SHPT). Previous studies have shown that there are different fragments of PTH in the body's circulation, causing antagonistic effects on bone and the kidney. Here we review the metabolism of PTH fragments; the progress being made in PTH measurement assays; the effects of PTH fragments on bone, kidney, and the cardiovascular system in CKD; and the predictive value of PTH measurement in assessing the effectiveness of parathyroidectomy (PTX). We hope that this review will help to clarify the value of accurate PTH measurements in CKD-MBD and promote the further development of multidisciplinary diagnosis and treatment.

## 1. Introduction

Chronic kidney disease-mineral and bone disorder (CKD-MBD) is a complication of CKD characterized by dysregulated mineral and bone metabolism, bone abnormalities, and vascular calcification, all which contribute to cardiovascular disease (CVD) and mortality [1]. Parathyroid hormone (PTH) reflects the function of the parathyroid gland and also takes part in the metabolism of minerals. In circulation, there are different fragments of PTH, including (1-84)PTH and (7-84)PTH [2–4]. (1-84)PTH is also named as whole PTH (wPTH). It has been reported that circulating (7-84)PTH can antagonize the biological activation of (1-84)PTH in bones and kidneys [5–7]. Currently, the second-generation intact parathyroid hormone (iPTH) assay is the most commonly used method for measuring PTH levels. The iPTH assay detects both full-length (1-84)PTH and (7-84)PTH fragments, while third-generation PTH assays are specific for (1-84)PTH [8, 9]. Accurate detection of blood PTH levels is crucial for evaluating parathyroid function and for the clinical management of CKD-MBD patients. In this review, we summarize the metabolism of PTH fragments; the progress being made in PTH measurement assays; the effects of PTH fragments on bone, kidney, and the cardiovascular system in CKD; and the predictive value of PTH measurement in assessing the effectiveness of parathyroidectomy (PTX).

## 2. Characteristics of PTH Metabolism

**Figure 1:**
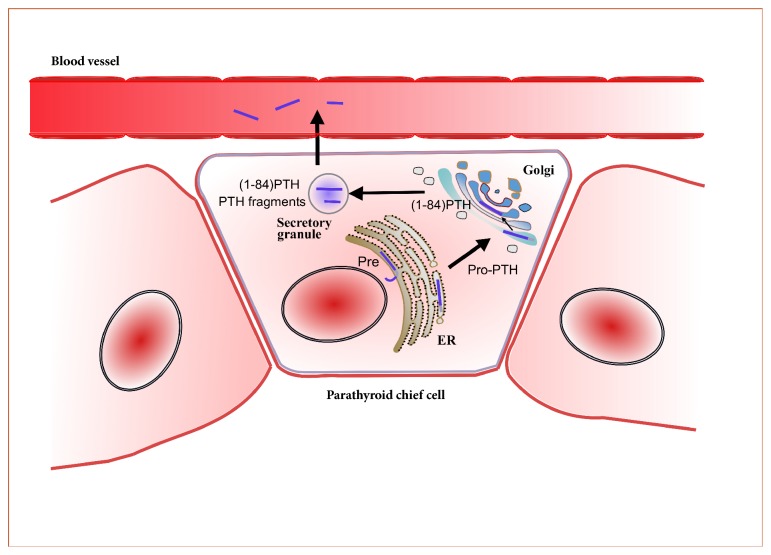
PTH production and secretion.

PTH is a single-chain hormone of 84 amino acids produced mainly by chief cells in the parathyroid glands; it has a relative molecular weight of 9.5 kD. PTH is cleaved from pre-pro-PTH, consisting of 115 amino acids, to pro-PTH, consisting of 90 amino acids [10]. The latter subsequently splits into active (1-84)PTH, which is stored in secretory granules (Figure 1). After secretion, PTH is further metabolized into different fragments in the liver.

In the circulation, PTH is composed of full-length (1-84)PTH, amino-terminal PTH, carboxyl-terminal PTH, and middle-PTH [10, 11]; (1-84)PTH accounts for 18% of the circulating PTH; long C-PTH, including (7-84)PTH, (10-84)PTH, and (15-84)PTH, represents only about 5% [12]. Like other C-PTH fragments, (7-84)PTH can be both degraded from (1-84)PTH in the liver and secreted by the parathyroid glands [13]. These fragments accumulate in the circulation of CKD patients and account for up to 45-50% of all circulating PTH molecular forms because they are mainly cleared through the kidney [14]. Previous studies have demonstrated that (7-84)PTH and C-PTH fragments increase as the estimated glomerular filtration rate (eGFR) decreases [15].

The synthesis and secretion of PTH are mainly regulated by the extracellular calcium (Ca^2+^) level. Extracellular calcium binds and activates the calcium-sensing receptor (CaSR) on the parathyroid cells, leading to a reduced PTH release. Hypercalcemia not only reduces overall PTH secretion but also contributes to the release of PTH fragments, whereas hypocalcemia stimulates overall PTH secretion and favors the release of (1-84)PTH [16, 17].

**Figure 2:**
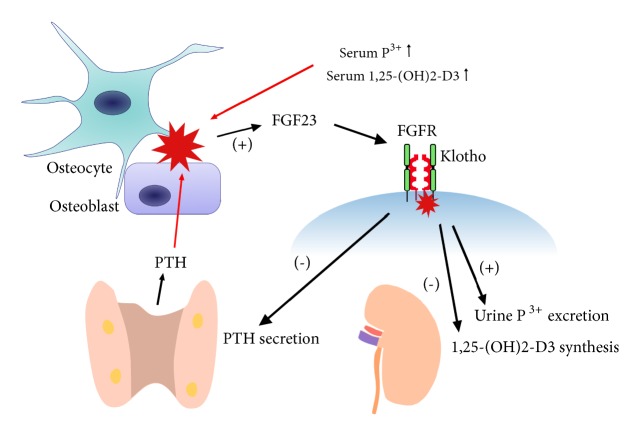
Interactions between FGF23 and PTH.

In addition to calcium, there are other factors that regulate PTH secretion under physiological conditions. High phosphorus concentrations stimulate PTH secretion, although the mechanism remains unknown [18]. Calcitriol is mainly synthesized by kidneys through the hydroxylation of calcidiol, which is mediated by the enzyme 1*α*-hydroxylase. PTH can enhance the production of 1,25(OH)_2_D_3_, and in turn 1,25(OH)_2_D_3_ inhibits the production of PTH by either directly affecting the transcription level of the PTH gene or indirectly increasing Ca^2+^ absorption in the intestine and stimulating the expression of CaSRs [19]. Fibroblast growth factor 23 (FGF23) is secreted primarily by osteoblasts and osteocytes in response to increased serum phosphorus or 1,25(OH)_2_D_3_. FGF23 not only targets the parathyroid gland to decrease PTH secretion, but also targets the kidney to increase urinary phosphorus excretion and suppress synthesis of 1,25(OH)_2_D_3_ [20]. These effects are mediated through activation of the FGF receptor (FGFR) and its coreceptor klotho. PTH also increases FGF23 production which enhances phosphaturia and reduces renal production of vitamin D. It is believed that FGF23 and PTH mutually regulate each other in a negative feedback loop, where PTH stimulates FGF23 production and FGF23 in turn suppresses PTH synthesis [21]. The interactions between FGF23 and PTH were shown in Figure 2. FGF23 levels increase early in the course of CKD which occur long before evident changes in serum phosphate and calcium levels. FGF23 should be a kidney function-dependent surrogate for another risk factor after adjusting important covariates including phosphate, calcitriol, and vitamin D therapy [22]. Thus, besides PTH, measuring the levels of FGF23 should be helpful in improving clinical management of CKD-MBD patients. In summary, calcium and phosphorus together with the 1,25(OH)_2_D_3_ and FGF23 play complex roles in homeostasis of PTH via positive and negative feedback loops, which are important in the pathogenesis of CKD-MBD [19].

## 3. Progress and Application of PTH Assays

Radioimmunoassay (RIA), the first-generation assay for measuring PTH, was described in 1963 [31]. It used a single antibody to recognize (1-84)PTH, abundant inactive middle-PTH, and C-PTH fragments. This assay has poor specificity and sensitivity, which means that it cannot detect low PTH levels. It is therefore no longer used in clinical practice.

In 1987, the second-generation iPTH assay was developed and applied by Nichols Institute Diagnostics [29]. It used a double monoclonal antibody, the capture antibody being directed toward the 39-84 portion of C-PTH and the labeled antibody reacting to the 13-24 portion of N-PTH. For a long time, the second-generation iPTH assay was believed to react only with full-length (1-84)PTH. Later studies, however, showed that it was reacting with both (1-84)PTH and long C-PTH fragments, mainly (7-84)PTH [8].

The first third-generation assay was launched in 1999. It used an anti-C-terminal antibody similar to that of the second-generation iPTH assay. However, an anti-N-terminal antibody directed toward the very first amino acids (1-4) of the peptide had no reaction to the (7-84)PTH fragment [8]. An immunoradiometric assay (IRMA) that was used to assay (1-84)PTH had initially been restricted to clinical use owing to the effects caused by human manipulation, longer measurement time, and radioactive pollution. More recently, a new (1-84)PTH assay with a fully automatic electrochemiluminescence immunoassay (ECLIA) has come into clinical use [32].

Non-(1-84)PTH accounts for 20% of iPTH in people with normal kidney function, however, increases to 50% in hemodialysis patients [11]. Unlike other inactive short C-PTH fragments, (7-84)PTH can antagonize the biological activity of (1-84)PTH in the skeleton and kidney [33, 34]. It is showed that the diagnosis by intact PTH accorded with that by (1-84)PTH in over 90% of the control group, supporting the conventional concept that parathyroid function could be assessed by either the intact PTH or the (1-84)PTH [13]. As the circulating PTH levels increased, there was a large difference between the iPTH and (1-84)PTH assays [17]. Twenty-eight percent of the total population was misclassified within an iPTH target range of the Japanese Society for Dialysis Therapy (JSDT) guidelines [35], thus the (1-84) PTH assay will be more useful for precise evaluation of PTH activity than the iPTH assay [17]. Furthermore, Koda et al. [13] discovered that there were large differences between circulating levels of iPTH and (1-84) PTH in those SHPT patients treated with cinacalcet. Cinacalcet, binding to the calcium-sensing receptor, directly suppressed parathyroid function and stimulated degradation rate of (1-84)PTH to PTH fragments including (7-84)PTH within the parathyroid gland, resulting in significant overestimation and subsequent overtreatment when evaluating parathyroid function with intact PTH according to the clinical practice guidelines [13, 28]. Although the second-generation assay may overestimate the severity of hyperparathyroidism in patients with CKD, especially in individuals with severe SHPT, it still is recommended for detecting circulating PTH levels by the KDIGO guidelines [36], probably because currently available clinical evidence about (1-84) PTH is rather limited compared to that of iPTH values.

Some studies are showing that (1-84)PTH or the PTH ratio, such as (1-84)PTH/iPTH and (1-84)PTH/(7-84)PTH, are superior to single iPTH values for evaluating bone turnover in CKD patients, predicting the effectiveness of PTX, and estimating the fatality rate among hemodialysis patients [24, 28, 37, 38]. It was reported that higher (1-84)PTH/iPTH ratio, but not serum (1-84)PTH and iPTH, predicted higher all-cause mortality in male hemodialysis patients [28]. However, the third-generation PTH assays also have a drawback in that they can detect the levels of N-PTH fragments in serum. N-PTH has a modified 15-20 region and is less reactive in the second-generation PTH assays [39]. The third-generation assay used an anti-C-terminal antibody similar to those of the second-generation intact PTH assay and an anti-N-terminal antibody which directed toward the very first amino acids(1-4) of the peptide had no reaction to (7-84)PTH fragment. It means that the labeled antibodies of this assay can only recognize the 1-4 portion and thus may bind both (1-84)PTH and N-PTH but not the (7-84)PTH [40]. The theoretical value of (1-84)PTH/iPTH should be less than 1. It has been found to be about 0.4 to 0.7 in CKD patients [26, 41]. However, several cases have recently been reported where this value was more than 1; these generally involved patients with parathyroid carcinoma, severe SHPT, or primary hyperparathyroidism (PHPT) [42]. The abnormal ratio may be related to circulating high N-PTH levels, indicating that N-PTH can also be detected by the third-generation PTH assays [26, 43]. But the biological activity of N-PTH fragments and the mechanisms involved are still not clear.

**Figure 3:**
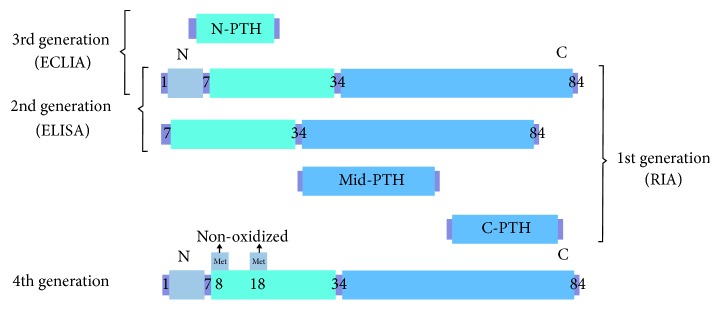
Methods of assaying PTH fragments.

At present there is no specific assay to detect (7-84)PTH; according to most researches [11, 25, 44], circulating (7-84)PTH levels are calculated indirectly subtracting the (1-84)PTH value from the iPTH value. Circulating (7-84)PTH acquired by subtraction is actually composed of various long C-PTH fragments, including biologically active (7-84)PTH and inactive long C-PTH fragments such as (10-84)PTH and (15-84)PTH. Methods for assaying PTH fragments are shown in Figure 3.

Obviously, the currently used PTH assays (the second- and third-generation PTH assays) make great progress in measuring bioactive PTH compared to the first-generation PTH assay. However, these methods ignore a second biologic process altering the native (1-84)PTH (other than PTH fragmentation): PTH oxidation. (1-84)PTH has two methionine amino acids at positions 8 and 18 within the receptor binding site that can be oxidized in vivo [45].

PTH can be oxidized in patients with renal disease, and oxidation of PTH at methionine residues 8 and/or 18 results in loss of biological activity. Thus, the biological properties of oxidized and nonoxidized PTH (n-oxPTH) are substantially different. Since oxidized PTH (oxPTH) is biologically inactive, the currently used methods to detect PTH in daily clinical practice may not adequately reflect PTH-related bone and cardiovascular abnormalities in patients on dialysis. The fourth-generation assay, namely the method of detecting nonoxidizing PTH, removes all oxidized forms of (1-84)PTH in plasma by targeting the antibody at the oxidation site, and then measures it by capturing and detecting antibody [46]. Tepel et al. had demonstrated that measurements of n-oxPTH reflected the hormone status more precisely [47]. Another study also had found that a huge proportion of circulating PTH was oxidized and thus not biologically active by using sensitive mass spectroscopy approaches to measure nonoxidized PTH in 1564 patients with chronic renal failure. And clinical studies demonstrated that bioactive, n-oxPTH, but not iPTH nor oxPTH, was associated with mortality in CKD patients [48]. It was shown that n-oxPTH levels were generally low relative to iPTH, and increased levels of n-oxPTH were related to decreased mortality in dialysis patients [47]. In a EVOLVE trial of 2,867 participants with follow-up of 64 months, the authors found that n-oxPTH, but not oxPTH or iPTH, was associated with the EVOLVE primary composite endpoint (time until death, myocardial infarction, hospitalization for unstable angina, heart failure, or a peripheral vascular event; hazard ratio 1.078; 95% CI 1.012-1.148;* p*=0.020), cardiovascular mortality (hazard ratio 1.111; 95% CI 1.014-1.218;* p*=0.024), and all-cause mortality (hazard ratio 1.113; 95% CI 1.038-1.193;* p*= 0.003) [48, 49]. However, n-oxPTH assay is not ready for clinical use and still needs further exploration.

## 4. Predictive Value of Measuring PTH Fragments during the PTX Perioperative Period

SHPT, a complication of CKD-MBD, is associated with multiple systemic manifestations and contributes to adverse outcomes. PTX is an effective treatment for patients with severe SHPT who cannot be helped with medical therapy [50, 51]. The number and locations of the parathyroid glands play an important role in determining the success of surgical treatments. Most people have four parathyroid glands (~90.4%). However, 2.4% have three and 7.2% have more than four. Ectopic parathyroid glands are usually located around the thymus, mediastinum, carotid sheath; in SHPT patients, the frequency of ectopic parathyroid glands is around 4.2% [52]. Supernumerary and ectopic parathyroid glands make it difficult to perform successful PTX surgery because if these glands are not properly resected, SHPT will persist, which occurred in 0.4% to 26% as reported [53–55]. Imaging of parathyroid glands before PTX is helpful to guarantee the safety and efficacy of treatment. However, the sensitivity of ultrasonography (USG) and 99mTc-sestamibi (MIBI) which are most commonly used diagnostic imaging techniques in SHPT patients was 54%-91.5% and 25%-62% respectively [56, 57]. In view of these facts, the precise detection of a patient's serum PTH level during the perioperative period can help to confirm that all of the parathyroid glands have been removed [58]. Such confirmation would make it possible to avoid extra exploration; it would also decrease the risk of surgical complications and help to determine whether the surgery was successful.

The half-lives of iPTH, (1-84)PTH, and (7-84)PTH are not the same. In a study of 77 PHPT patients with a single adenoma, Yamashita et al. reported that the half-lives of iPTH, (1-84)PTH, and (7-84)PTH were 2.92 ± 0.13, 2.33 ± 0.09, and 9.89 ± 3.30 minutes, respectively [59]. In another study by the same group of 28 SHPT patients who underwent total PTX with autotransplantation, the half-lives of iPTH, (1-84)PTH, and (7-84)PTH were shown to be 8.1 ± 0.6, 4.6 ± 0.4, and 24.0 ± 15.8 minutes, respectively [60]. This research shows that the half-lives of PTH fragments are significantly longer in patients with kidney dysfunction. Furthermore, the half-life of (1-84)PTH is the shortest among all the PTH fragments, while that of (7-84)PTH is the longest. Our previous research showed that >88.9% decline of serum iPTH values at 20min after removing the last parathyroid gland could predict successful PTX (sensitivity 78.6%, specificity 88.5%) [61]. Susumu's study pointed to a decline of more than 89.29% in (1-84)PTH values at 10 minutes after the last parathyroid gland was removed, and this confirmed the success of PTX (sensitivity 100% specificity 90%) [62]. Moreover, measurement using the third-generation PTH assay with simple manipulation takes about 18 minutes. Thus (1-84)PTH with its shorter half-life is more appropriate than an iPTH assay for predicting surgical outcome of PTX [24, 59, 60].

## 5. The Biological Function of Parathyroid Hormone

Classic PTH exerts multiple actions by binding its biologically active domain in the first 34 amino acids of the N-terminal to PTH type 1 receptor (PTHR1). PTHR1 is mainly expressed in bone and kidney but also at lower levels in other tissues. Nevertheless, (7-84)PTH may play its biological role by combining with C-PTH receptor (C-PTHR), which is mainly expressed in osteoblasts and osteocytes and can antagonize the effect of the PTH/PTHR1 system after activation.

### 5.1. Effects of PTH Fragments on the Skeleton

PTH regulates bone remodeling through its direct actions on osteoblasts and osteocytes and indirect actions on osteoclasts. PTH induces bone formation by downregulating the expression of sclerostin in osteocytes, thus permitting the anabolic Wnt signaling pathway to proceed. By combining with its receptor in osteoblasts and osteocytes, PTH can increase the receptor activation of the nuclear factor-kappa B ligand (RANKL)/osteoprotegerin (OPG) ratio and osteoclast activity, thereby stimulating bone resorption [63]. Although PTH stimulates both the formation and resorption of bone, its effect on bone mass depends mainly on the duration of exposure and dose of PTH [63, 64]. Successive administration of PTH increases osteoclast activity. By contrast, intermittent low-dose administration of PTH can increase osteoanabolic activity [64].

Disturbances of bone and mineral metabolism in patients with CKD can be defined as disorders of high, normal, and low bone turnover. PTH has been the noninvasive biomarker to estimate the parathyroid function and bone turnover state of the patients with CKD although bone biopsy is a gold standard for the judgement of bone turnover [41, 44, 65]. A study has shown that the (1-84)PTH/(7-84)PTH ratio can predict bone turnover. A ratio greater than 1 suggests high or normal bone turnover (sensitivity 100%) while a ratio of less than 1 indicates low turnover (sensitivity 87.5%) [37, 66]. Specific (1-84) PTH assays neglect the role of (7-84) PTH, which is to inhibit bone turnover. According to independent bone biopsy studies, the (1-84)PTH/(7-84)PTH ratio is 94% accurate in identifying adynamic bone disease and 94% accurate in assessing bone turnover status [67].

**Figure 4:**
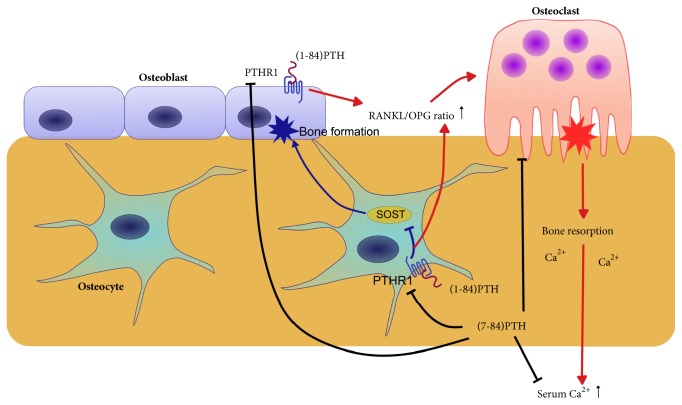
Effects of (1-84)PTH and (7-84)PTH on skeleton.

Increased osteoblastic and osteoclastic activity is consistent with increased PTH levels in people with normal kidney function. On the other hand, the skeleton in long-term hemodialysis patients with high bone turnover disease is resistant to the actions of PTH, which may be relevant to the heavy accumulation of (7-84)PTH [68]. There are two possible pathogeneses to account for this phenomenon. First, (7-84)PTH could decrease the effect of (1-84)PTH on plasma calcium. When (1-84)PTH and (7-84)PTH were given simultaneously in a 1:1 molar ratio to parathyroidectomized rats, the calcemic response to (1-84)PTH was decreased by 94% (*P* <.001) [23]. The second possibility is that (7-84)PTH could reduce the expression of PTHR1 in skeleton by endocytosis of the receptor or decrease bone turnover by inhibiting osteoclastic activity independent of PTHR1 [69, 70]. The effects of (1-84)PTH and (7-84)PTH on the skeleton are shown in Figure 4.

### 5.2. Effects of PTH Fragments on the Kidney

**Figure 5:**
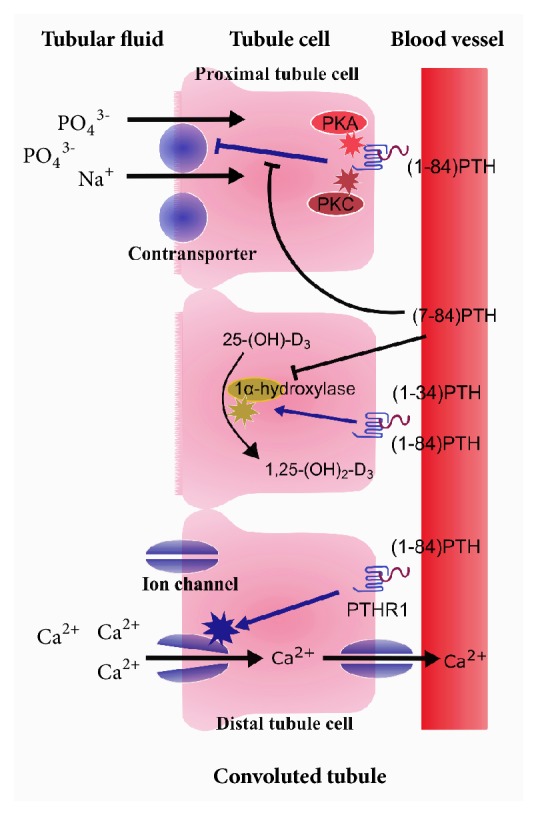
Effects of (1-84)PTH and (7-84)PTH on kidney.

PTH plays an important role in the kidney. It promotes Ca^2+^ reabsorption by stimulating the specific ion channels of the distal convoluted tubule and increases urinary phosphate excretion by regulating sodium-phosphate cotransporters in the proximal convolute tubule through protein kinase A- and C-dependent pathways. iPTH also indirectly increases intestinal calcium and phosphorus absorption by stimulating renal 1*α*-hydroxylase activity, which can convert 25-(OH)-vitamin D to 1,25(OH)_2_D_3_ [71]. Nakajima et al. [5] investigated primary cultured murine renal tubules cells and found that (1-34)PTH stimulated the conversion of 25-(OH)-vitamin D to 1,25(OH)_2_D_3_, whereas (7-84)PTH dosing dependently antagonized this process. Apart from the effects on plasma calcium above, Slatopolsky et al. [23] also found that (7-84)PTH decreased phosphaturic excretion by 50.2% (*P* <0.05) when (1-84)PTH and (7-84)PTH were given simultaneously to normal rats (Figure 5).

The response to PTH is decreased in the patients with CKD. Multiple factors are involved, including increasing levels of circulating phosphate, deficient calcitriol, oxidative stress, decreased PTHR1 expression, and the function of accumulated PTH fragments, especially heavily accumulated (7-84)PTH [16].

## 6. Effects of PTH Fragments on Cardiovascular Disease and Mortality in CKD Patients

CVD is a common complication and the leading cause of death in patients with CKD. Those with CKD and end-stage renal disease are at 5 to 10 times greater risk for CVD than healthy people [72]. According to the latest information from the US Renal Data System, the incidence of CVD in CKD patients over 66 years of age is as high as 69.8% [73]. Studies have shown that CVD and mortality in hemodialysis patients are related to abnormal levels of PTH [74–76], which are closely related to the disordered autonomic nerve function [77].

**Figure 6:**
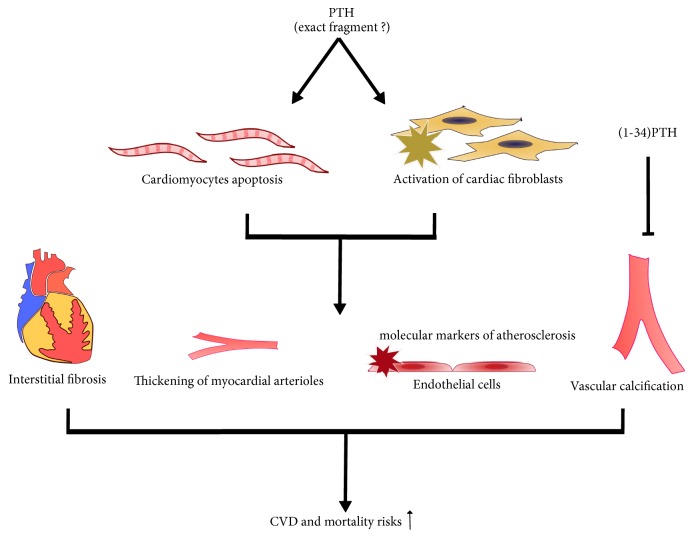
Effects of PTH on the cardiovascular system.

The researches have demonstrated PTH receptors in the cardiovascular (CV) system such as cardiomyocytes, vascular smooth muscle, and endothelial cells [78], which indicates that inappropriate PTH secretion may impact on the CV health including the induction of apoptosis in cardiomyocytes; activation of cardiac fibroblasts, thus inducing interstitial fibrosis; thickening of the myocardial arterioles; increasing the endothelial expression of molecular markers of atherosclerosis, such as the receptor of advanced glycation end products and IL-6; and the triggering of vascular calcification in a phosphate-independent manner [27]. Myocardial fibrosis is a common CV complication at the end stage of CKD. It may be related to the increased PTH levels which can promote increased Ca^2+^ uptake into mitochondria of cardiomyocytes and smooth muscle cells. And the exposure of mitochondria to calcium overload and oxidative stress causes reduced intramitochondrial ATP levels, necrotic cell death, and eventually myocardial fibrosis [78], whereas Sebastian et al. [79] have reported that, in CKD rats, intermittent administration of (1-34)PTH decreased vascular calcification, while the same dosage of (7-34)PTH had little effect in this regard. This research also indicated that downregulating the PTH1R reduced not only bone demineralization but also vascular calcification. However, (7-34)PTH has a poor affinity for PTHR1 and may play a role at higher concentrations [79]. The effects of different PTH fragments on the cardiovascular system are shown in Figure 6.

## 7. Conclusion

**Figure 7:**
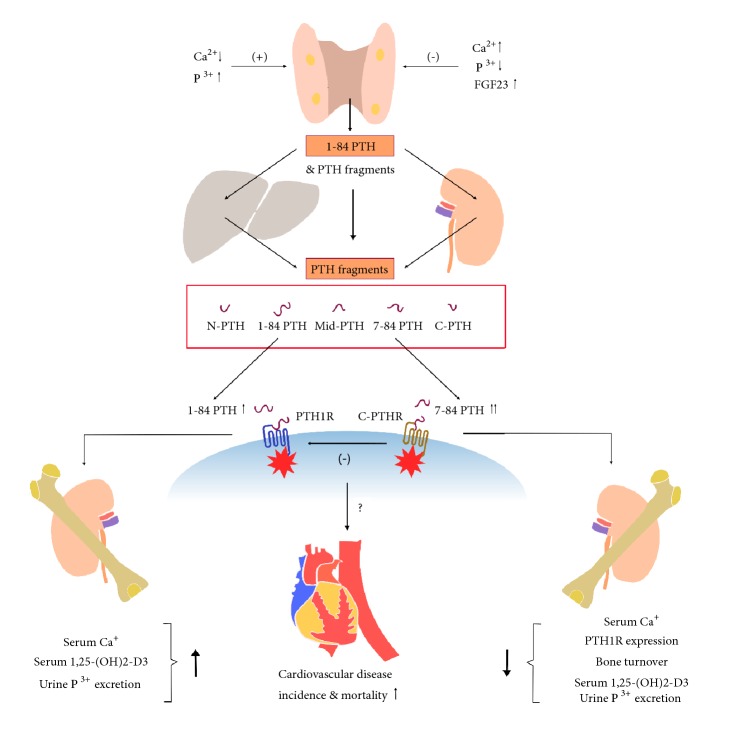
Metabolism and effects of different PTH fragments on multisystem in CKD patients.

PTH plays a vital role in regulating bone and mineral metabolism, and accurate measurement of PTH is an essential part of the clinical management of patients with CKD-MBD. As the most widely used method, second-generation iPTH assays detect not only full-length (1-84)PTH but also N-terminally truncated PTH, mostly (7-84)PTH, which has been regarded as biologically inactive or having antagonistic effects against (1-84)PTH on both bone and kidney. However, the effects of (1-84)PTH and (7-84)PTH on the cardiovascular system still require further exploration. We have summarized metabolism and effects of different PTH fragments on kidney, bone, and cardiovascular system in CKD patients (Figure 7). The different biological effects of PTH fragments are new targets for the accurate clinical diagnosis and treatment of CKD-MBD patients. A summary of key findings in studies analyzing PTH fragments clinically was shown in Table 1. The third-generation PTH assays may be superior for evaluating bone turnover in patients with CKD, estimating the success of PTX, and predicting CVD and mortality in CKD patients. Nevertheless, the clinical application and guide value of the PTH fragments await further clinical and basic investigations.

## Figures and Tables

**Table 1:** The summary of key findings in studies analyzing PTH fragments clinically.

Year	Number of patients	Study type	Methodology	Key findings
2000 [23]	Uremic patients (n = 28) and renal transplant patients (n =14)	Cross-sectional study	(1-84) PTH was detected by a new IRMA assay and iPTH assay was purchased from the Nichols Institute (I-Nichols, San Juan Capistrano, CA, USA).	In CKD patients, the presence of high circulating levels of non-(1-84) PTH fragments (most likely (7-84) PTH) detected by the “intact” assay and the antagonistic effects of (7-84) PTH on the biological activity of (1-84) PTH explain the need of higher levels of “intact” PTH to prevent adynamic bone disease.

2004 [24]	PHPT patients (n = 74) and SHPT patients (n =18) who underwent PTX	Cross-sectional study	iPTH and (1-84) PTH were detected both by 2-site immunochemiluminometric assay.	Plasma (1-84) PTH decreased more rapidly than iPTH after PTX in patients in both the PHPT and SHPT groups, which suggested that a quick (1-84) PTH assay may be a more useful adjunct to PTX in both SHPT and PHPT.

2008 [25]	Dialysis patients (n =515)	Cohort study	Scantibodies Clinical Laboratory (SCL; Santee, CA, USA) was responsible for measuring iPTH and (1-84) PTH, no detailed methodology was discussed.	The circulating levels of (1-84) PTH and iPTH were highly correlated. Elevated levels of (1-84) PTH was significantly associated with increased mortality, whereas iPTH did not reach statistical significance.

2010 [26]	A SHPT patient with cinacalcet therapy	Case report	Detailed methods for measuring iPTH and (1-84) PTH were not mentioned.	This patient had abnormally higher (1-84) PTH levels than iPTH levels and (1-84) PTH/iPTH ratio was reversed by cinacalcet therapy.

2011 [9]	Hemodialysis patients (n =53)	Cohort study	Elecsys iPTH and (1-84) PTH assay were measured by ECLIA, and Whole PTH assay was measured by IRMA.	The Elecsys (1-84) PTH assay provides comparable data to the Whole PTH assay for monitoring parathyroid function in patients receiving hemodialysis.

2011 [15]	Patients with varying stages of CKD (n =203)	Cross-sectional study	iPTH and (1-84) PTH were measured both by immunoassay.	iPTH, (1-84) PTH, and (7-84) PTH increase with increasing CKD stages, with a relatively greater increase in (7-84) PTH.

2011 [17]	Hemodialysis patients (n =738)	Cross-sectional study	(1-84)PTH was detected by immunoradiometric assay and method for measuring iPTH was not mentioned.	As the circulating PTH levels increased, there was a large difference between the iPTH and (1-84) PTH assays. Twenty-eight percent of the total population was misclassified within an iPTH target range of the Japanese guidelines.

2011 [27]	Hemodialysis patients (n =70)	Cohort study	iPTH and (1-84) PTH were measured by Nichols Advantage Intact PTH and Nichols Bio-Intact PTH Chemiluminescence Assays respectively.	A higher (1-84) PTH /non-(1-84) PTH ratio is associated with an increased risk for cardiovascular events in hemodialysis patients.

2013 [28]	Male hemodialysis patients (n =177)	Cohort study	iPTH was measured by electrochemiluminescence immunoassay, and (1-84) PTH was detected by two-site IRMA assay.	The higher group in (1-84) PTH/iPTH ratio had significantly higher all-cause mortality than the lower group.

2014 [11]	Patients on peritoneal dialysis (PD) (n =73)	Cross-sectional study	PTH was quantified by six second generation assays (one isotopic and five chemiluminescence assays) and by one third generation PTH method (IRMA assay).	PD patients have a higher proportion of (7-84) PTH circulating fragments than hemodialysis patients assessed previously.

2015 [13]	Adult dialysis patients with cinacalcet therapy (n =44) and without (n =112)	Cohort study	iPTH was detected by an electrochemiluminescence immunocomparable assay, and (1-84) PTH was detected by an immunoradiometric assay.	The (1-84) PTH/iPTH ratio in cinacalcet users is lower than that in the non-users with comparable levels of serum Ca.

2017 [29]	Hemodialysis patients (n =145)	Cohort study	iPTH and (1-84) PTH were both detected by an electrochemiluminescence method.	Didn't find any advantages to using (1-84) PTH vs. iPTH as a marker of mortality. (1-84) PTH limits of normality must be reevaluated because its relationship with iPTH is not consistent.

2018 [30]	Stage 5 CKD patients (n =262) including those who underwent PTX (n =92)	Cross-sectional and cohort study	iPTH and (1-84) PTH were both measured by ECLIA method.	Stage 5 CKD patients had higher plasma levels of different PTH fragments, and lower (1-84) PTH/iPTH ratio. PTX could significantly reverse these abnormalities in severe SHPT patients.
